# Neurosciences and Sports Rehabilitation in ACLR: A Narrative Review on Winning Alliance Strategies and Connecting the Dots

**DOI:** 10.3390/jfmk10020119

**Published:** 2025-04-02

**Authors:** Rocco Salvatore Calabrò, Andrea Calderone, Nicola Fiorente

**Affiliations:** 1 IRCCS Centro Neurolesi Bonino-Pulejo, S.S. 113 Via Palermo, C.da Casazza, 98124 Messina, Italy; andrea.calderone95@gmail.com; 2 Polimedica Fisio&Sport, 35013 Cittadella, Italy; nicola.fiorente@gmail.com

**Keywords:** sport medicine, neurorehabilitation, ACL, muscle strength, neuroplasticity, neuroscience

## Abstract

This narrative review explores the significant evolution of sports rehabilitation, tracing its trajectory from basic exercise therapies of the early 20th century to the advanced, neuroplasticity-driven approaches of the 21st century, with a specific focus on anterior cruciate ligament reconstruction (ACLR). The primary aim is to understand how neuroplasticity, motor control, and sensorimotor retraining can optimize recovery, reduce reinjury risk, and enhance long-term athletic performance, and to synthesize current rehabilitation strategies that integrate innovative technologies, such as robotics, virtual reality (VR), and biofeedback systems, to address the neurocognitive deficits that contribute to the alarmingly high reinjury rates (9–29%) observed in young athletes post-ACLR. These deficits include impaired proprioception, motor control, and psychological factors like fear of reinjury. The methodology employed involves a narrative review of peer-reviewed literature from databases including PubMed, Scopus, and Web of Science. The synthesis of findings underscores the importance of holistic rehabilitation approaches, including targeted proprioceptive exercises, dual-task drills, and immersive VR training, in enhancing sensorimotor integration, decision-making, and athlete confidence. Furthermore, this review highlights the critical need for long-term monitoring and interdisciplinary collaboration between neuroscientists, physiotherapists, and engineers to refine rehabilitation protocols and ensure sustained recovery. By leveraging neuroplasticity and advanced technologies, the field can shift from a focus on purely physical restoration to comprehensive recovery models that significantly reduce reinjury risks and optimize athletic performance.

## 1. Introduction

Rehabilitation strategies have been subject to a deep evolution of sports medicine over the past century, paralleling advancements in neuroscience, biomechanics, and technology [1]. Early in the 20th century, exercise therapy for sports injuries was largely confined to general hospital settings, where interventions were primarily focused on passive mobilization and basic strength training [2]. These early regimens, while rudimentary by today’s standards due to limited understanding of biomechanics, physiology, and specific injury pathologies, were instrumental in establishing foundational rehabilitation principles centered on movement restoration, progressive loading, and functional reintegration [3]. Although simple by today’s standards, lacking the sophisticated diagnostic tools, evidence-based protocols, and technology-driven interventions now commonplace, these treatments provided the foundation for informed specificities to the treatment of sports injuries [4]. Over time, as clinical observations and empirical data accumulated, sports rehabilitation evolved into a more structured discipline, integrating targeted exercise protocols and injury-specific rehabilitation models [5,6,7]. The recognition of biomechanical factors in injury prevention and recovery further refined rehabilitation practices, paving the way for evidence-based strategies tailored to individual needs [8]. Simultaneously, the field of neurorehabilitation was undergoing a parallel evolution. Initially, neurorehabilitation strategies were largely empirical, borrowing exercises from general physiotherapy and even adapting rudimentary movement-based interventions inspired by animal rehabilitation practices [9]. However, by the mid-20th century, increasing awareness of the central nervous system’s role in recovery led to more specialized therapeutic approaches [8]. Breakthroughs in neuroscience, particularly in understanding motor control and neural adaptation, catalyzed a shift toward structured neurorehabilitation protocols [9]. Specifically, advancements in motor control research illuminated the intricate interplay between the central nervous system, muscles, and sensory feedback, revealing how targeted exercises could optimize movement efficiency, coordination, and precision [10,11]. This included a deeper understanding of feedback loops, feedforward mechanisms, and the role of specific brain regions like the cerebellum and motor cortex in movement execution and learning [12,13,14]. A significant breakthrough occurred with the late 20th-century revelation of neuroplasticity, a remarkable characteristic of the brain to rearrange and establish new neural pathways in reaction to injury or training stimuli [15,16,17]. According to neuroscience, neuroplasticity is a major feature of the nervous system that allows it to adapt to internal and external stimuli by changing its structure, function, or connections [18]. This includes various processes, including synaptic plasticity (changes in the strength of neuron connections), neurogenesis (the production of new neurons), and changes in gene expression [19]. By comprehending how the brain responds to exercise and injury, sports rehabilitation programs can be developed to encourage neuroplastic adjustments that improve motor skills, coordination, and strength [20]. This may involve targeted exercises, cognitive training, and other interventions that stimulate specific brain regions and neural pathways [21]. For example, after an injury, the brain may need to find new pathways to execute movements that were previously automatic. Neuroplasticity allows for this rewiring, helping athletes regain lost function. This change in approach highlighted the significance of intense, repetitive, and task-focused training in promoting neural recovery, resulting in the creation of specialized neurorehabilitation facilities designed to tackle issues like stroke, spinal cord injuries, and neurodegenerative diseases [22,23]. These centers focused on teamwork across disciplines, integrating physical therapy, cognitive training, and neuromodulation methods to enhance functional results [24,25]. As the 21st century began, the incorporation of new technologies transformed both neurorehabilitation and sports rehabilitation, connecting neuroscience with recovery in athletic performance [26]. Robotics and exoskeleton-assisted therapy have improved gait retraining by offering precision-controlled motions that enhance neuromuscular re-education [27,28,29]. Immersive VR has added a new aspect to rehabilitation, involving patients in engaging, multisensory experiences that enhance motor learning and compliance with rehabilitation guidelines [30,31,32,33]. Biofeedback systems, employing real-time electromyographic (EMG) and kinematic information, enable clinicians to adjust rehabilitation programs according to objective physiological reactions, promoting more tailored and efficient recovery methods [34,35,36]. Moreover, brain–computer interface technology has surfaced as a hopeful instrument, allowing people with significant neurological disabilities to recover motor control via direct interpretation of brain signals [37,38,39]. Together, these innovations emphasize the intricate relationship among neuroscience, rehabilitation techniques, and technological progress, underscoring the necessity of a multidisciplinary strategy for enhancing recovery [40]. In spite of these impressive advancements, considerable obstacles remain in sports rehabilitation. A significant worry is the elevated reinjury rate after ACLR, which remains a major obstacle to complete recovery and returning to play [41,42]. Research indicates that neuromuscular imbalances, changes in proprioception, psychological aspects like kinesiophobia, and inadequate rehabilitation methods lead to a heightened risk of reinjury, especially among elite athletes [43,44,45].

Moreover, even after utilizing advanced technologies such as robotics and exoskeleton-assisted therapy, immersive VR, and biofeedback systems, a critical challenge remains in ensuring sustained efforts and maintained improvements. For each of these modalities, achieving long-term functional gains requires diligent adherence to post-therapy protocols, consistent monitoring, and ongoing adaptation to prevent relapse and facilitate a successful return to play [46,47,48,49,50]. Furthermore, discrepancies in return-to-play protocols and the lack of standardized neuromuscular training programs exacerbate the problem, underscoring the need for a more integrated, neuroscience-driven rehabilitation approach. Through the incorporation of motor learning principles such as varied practice, which involves performing movements under different conditions to enhance adaptability [51]; contextual interference, which strategically mixes practice tasks to improve retention [52]; and feedback optimization, which focuses on providing precise and timely feedback to refine movement patterns [51], alongside cognitive-motor interactions, and sensorimotor retraining, researchers and clinicians can develop more comprehensive rehabilitation strategies that enhance recovery while also decreasing the chance of future injuries.

### Objective and Rationale

**Figure 1:**
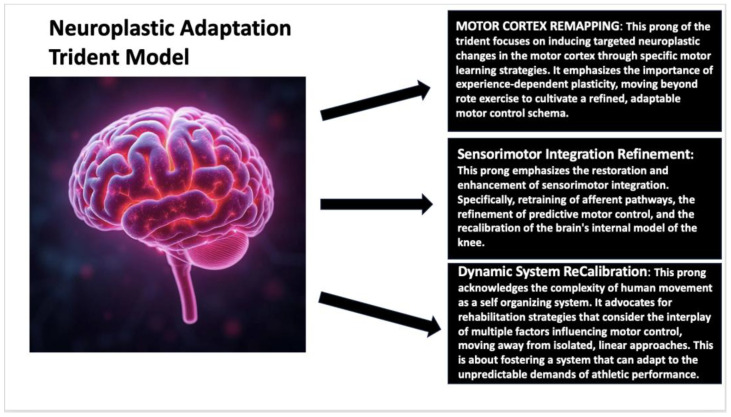
Neuroplastic Adaptation Trident Model.

This narrative review aims to synthesize current research on the integration of neuroscience and sports rehabilitation, specifically in ACLR. We focus on how neuroplasticity, motor control, and sensorimotor retraining can optimize recovery, reduce reinjury risk, and enhance long-term athletic performance, and synthesize current rehabilitation strategies that integrate innovative technologies, such as robotics, virtual reality (VR), and biofeedback systems, to address the neurocognitive deficits. The rationale for this review stems from persistent complications following rehabilitation for ACLR, including neuromuscular impairments, aberrant locomotive behavior, impairments in proprioception, and psychological impediments such as kinesiophobia [53]. Despite improvements in surgical interventions and rehabilitation protocols, the high reoccurrence of reinjuries continues to present a problem, and it is, therefore, imperative to implement a new, neuroscience-inspired model for post-ACLR rehabilitation. In this review, we introduce an innovative theoretical framework, which we call the Neuroplastic Adaptation Trident Model, to facilitate the incorporation of neuroscience principles into ACL rehabilitation [54]. This model suggests that the best recovery, minimized risk of reinjury, and improved long-term athletic performance are attained by concurrently and synergistically focusing on three essential neuroplastic adaptation pathways: Motor Cortex Remapping, Sensorimotor Integration Refinement, and Dynamic System Re-Calibration. The Neuroplastic Adaptation Trident Model suggests that by concurrently and strategically focusing on these three pathways, we can develop a more holistic and effective method for ACL rehabilitation, thereby improving patient results and reducing the likelihood of reinjury [54]. Figure 1 visually presents the characteristics of this model.

## 2. The Unwelcome Guest: ACL Reinjury Rates and Risk Factors

The prevalence of reinjury rates following ACLR ranges widely, from as low as 1.5% to as high as 37.5% [55,56,57]. Most studies report rates ranging from 9% to 29%, elevated in young, prepubertal males < 18 years of age relative to their female peers [58,59,60,61]. Notably, according to the study of Stracciolini et al., epidemiological data suggest that boys aged 5 to 12 years exhibit a greater proportion of ACL injuries relative to total sports-related injuries, whereas this trend shifts during adolescence, with girls experiencing a steeper rise in ACL injury prevalence post-puberty [62]. Apart from differences in age and sex, various other factors affect the risk of ACL reinjury. The interaction of biomechanics, neuromuscular regulation, and ligament healing ability is vital for post-ACLR results. Insufficient neuromuscular adaptation, lack of proprioception, and ongoing strength imbalances between the quadriceps and hamstrings can lead to reduced knee stability, raising the risk of reinjury [63,64]. Psychological elements, like fear of movement (kinesiophobia) and diminished self-confidence in high-pressure game scenarios, may additionally hinder return-to-play preparedness, especially in young athletes [65,66]. Additionally, genetic factors, ligament laxity, and previous ACL injuries on the opposite side have been proposed as possible risk modifiers affecting ACLR recovery paths [67,68]. These numbers and risk factors highlight the intrinsic hazards of high-impact sports like soccer and basketball, which require swift acceleration, abrupt deceleration, sudden directional shifts, and frequent powerful physical collisions [69]. The significant biomechanical pressures exerted on the lower limbs, especially the knee joint, subject athletes to high stress on the repaired ACL, frequently surpassing its ability to adapt [70]. Moreover, the unpredictable aspects of in-game situations (like unexpected tackles, awkward falls, and sudden pivots due to defensive pressure) significantly heighten the chance of reinjury. Moving forward, it is essential to explore how specific neuromuscular deficits, fatigue, and proprioceptive impairments contribute to the increased risk of reinjury in the post-ACLR rehabilitation phase.

### Risk Factors in Post-ACLR Rehabilitation: The Role of Neuromuscular Deficits, Fatigue, and Proprioceptive Impairments

**Figure 2:**
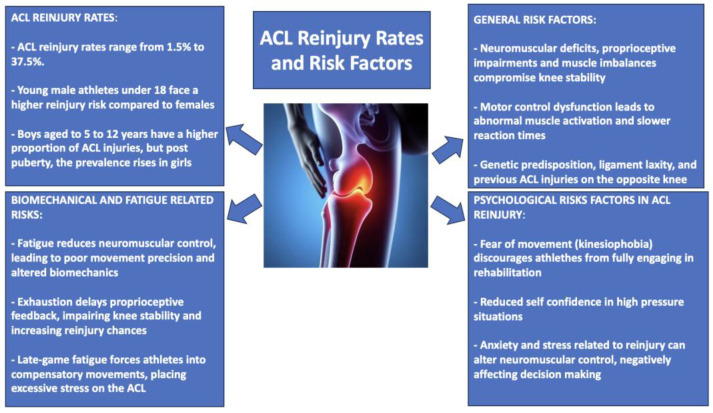
ACL reinjury rates and key risk factors.

Despite effective surgical reconstruction and rehabilitation, lingering neuromuscular deficits, slow reaction times, and uneven loading patterns may remain, increasing the knee’s susceptibility to excessive valgus stress and rotational instability. Additionally, fatigue can create a vicious cycle that significantly amplifies ACL injury risk. As athletes tire, their ability to control movements precisely deteriorates, leading to subtle yet critical changes in biomechanics [71]. This decline in neuromuscular control is compounded by a reduction in proprioceptive feedback, making it harder for athletes to sense and correct for these changes. The resulting compensatory movements, often subconscious, can place excessive strain on the ACL, making it more vulnerable to injury, especially during the high-stakes, late-game moments when exhaustion is at its peak [71]. The inadequacy of the usual rehabilitation is clear when viewed from the perspective of the complex requirements imposed on the athlete. Historically, however, these programs have emphasized the rehabilitation of physical structures (i.e., motion, strength, and endurance) [72]. Nevertheless, more recent studies have started to reveal understudied neurocognitive aspects in ACL reinjury, shifting focus beyond strength and biomechanics. Proprioceptive deficits impair joint awareness, leading to delayed neuromuscular responses and compromised stability [73]. Motor control dysfunction, including abnormal muscle activation and slower reaction times, increases stress on the knee, especially during high-intensity movements [74]. Athletes often develop excessive reliance on visual feedback, reducing adaptability in dynamic sports and delaying reflexive responses [75]. These impairments not only enhance reinjury risk but can also decrease athletes’ confidence and delay the return to optimal sports performance [76]. Considering these various risk factors, it is evident that conventional rehabilitation methods, although crucial, might be inadequate in tackling the fundamental neurocognitive and neuromuscular issues that lead to ACL reinjury. Therefore, it is essential to move towards more thorough rehabilitation methods, especially those that utilize the concepts of neuroplasticity to improve recovery and avert future injuries. Figure 2 provides an overview of ACL reinjury rates and key risk factors contributing to recurrence.

## 3. Reframing ACL Rehabilitation Through Neuroplasticity

Modern neuroscience offers an inspiring rationale for reframing ACL rehabilitation as a more holistic process. Neuroplasticity, the capacity of the brain to reorganize and create new neural connections, lies at the core of this reframing. An ACL injury does not merely disrupt the structural integrity of the ligament; it also interferes with the intricate communication between the knee and the brain [77]. This interference is reflected by changes in motor behavior and decreased sense of position in space and in fear of reinjury that does not allow one to regain confidence and participate with conviction in the recovery process [78,79]. To properly tackle these challenges, rehabilitation needs to stop thinking of the restoration of physical function as an isolated process. Proprioception, which is the capacity to detect joint position and motion, is essential in this process [80]. Reestablishing proprioceptive awareness is crucial for enhancing coordination and averting future injuries. To achieve this, rehabilitation plans must incorporate exercises that focus on the sensory feedback systems around the knee joint. For instance, exercising on unstable surfaces triggers sensory receptors near the knee, activating neural pathways and aiding in reestablishing communication between the brain and the knee [81]. This kind of training improves knee stability and also motivates the brain to create more natural, efficient movement patterns essential for the athlete’s ongoing success. Additionally, this neuroplastic change is essential for building the confidence required to participate in high-impact sports effectively and with diminished fear of reinjury [82]. Furthermore, cognitive difficulty is not to be underestimated as part of the rehabilitation process. While traditional rehabilitation approaches tend to focus predominantly on physical recovery, modern neuroscience acknowledges the indispensable role of the brain’s cognitive functions in facilitating a full recovery [83]. By introducing tasks that require athletes to perform multiple activities simultaneously (such as counting backward during drills or responding to unexpected stimuli), rehabilitation can address deficiencies in decision-making and reaction time [84]. These cognitive challenges reflect the real-world demands of athletic performance, where athletes must make rapid, adaptive decisions in the midst of high-pressure situations. These tasks aim to sharpen the athlete’s ability to react quickly and efficiently, simulating the fast-paced, dynamic environments they will face once they return to their respective sports. In sports such as soccer and basketball, where quick movements, sudden direction shifts, and regular physical contact are common, the strain on the body is significant, especially on the knee joint [85]. The strain on the repaired ACL is worsened by the unpredictable aspects of these games. Athletes are often confronted with situations that require split-second decisions, such as sudden pivots, tackling, or awkward landings, which can stress the knee beyond its current capacity for stability. Such conditions create an environment where the repaired ligament is highly susceptible to reinjury if the neuromuscular system and cognitive functions are not sufficiently prepared to handle the dynamic and chaotic nature of the sport [86]. This comprehension of neuroplasticity and the essential combination of cognitive and proprioceptive training lays the groundwork for the use of advanced technologies in ACL rehabilitation.

### 3.1. Next-Generation Rehabilitation: The Convergence of Robotics, VR, and Biofeedback in ACL Recovery

Contemporary sports rehabilitation incorporates cutting-edge technologies like robotics, VR, and biofeedback systems, which can improve neuroplasticity mechanisms throughout the recovery journey. Robotics, especially, serves as an effective resource for improving rehabilitation through exact, regulated movements that can aid in recovering typical motor function in a manner that conventional methods cannot achieve. Robotic devices like exoskeletons and robotic trainers can help athletes execute exercises with ideal biomechanics, facilitating a more focused and regulated rehabilitation process [87]. This regulated motion not only helps in building muscle and enhancing joint stability but also significantly contributes to retraining the brain to develop new motor skills. By delivering immediate feedback and modifying the intensity and nature of movement based on the person’s advancement, robotic systems present a tailored method for rehabilitation that enhances recovery [88,89]. Moreover, robotic rehabilitation tools can replicate the intricate movements needed in sports, enabling athletes to progressively recover the flexibility and agility essential for their particular athletic requirements [90]. By enhancing the neuromuscular control required for high-impact activities, robotic systems aid in the physical rehabilitation of the knee and promote the brain’s adjustment to new movement patterns, greatly improving recovery results [91]. For example, robotic-assisted therapy can improve walking speed and reduce time in functional tests like the 10-Meter Walk Test (10MWT) and Timed Up-and-Go (TUG) in athletes with ACLR. Moreover, the significant improvements in muscle strength, particularly in the vastus medialis, as measured by surface electromyography, suggest that robot-assisted rehabilitation not only promotes the restoration of functional movements but also facilitates muscle recovery at a deeper neuromuscular level, as demonstrated by the study of Hu et al. [92]. Moving forward, VR technology represents another promising new frontier in ACL rehabilitation. By immersing patients in interactive and engaging virtual environments, VR addresses several key challenges associated with recovery. Firstly, VR significantly enhances patient engagement and motivation [93]. Unlike repetitive and potentially monotonous physical therapy exercises, VR transforms rehabilitation into an interactive and enjoyable experience. Immersive VR environments provide a scripted but richly interactive environment in which athletes can train to react to unpredictable threats. VR, by activating the brain with scenario-based complex training, induces neural changes that lead to improved movement control and coordination [94]. Furthermore, VR can have a notable effect on knee moment (*p* < 0.001), knee flexion excursion (*p* = 0.03), and knee angle at peak vertical ground reaction force (*p* = 0.01). Significantly, ACLR patients in the VR condition showed knee joint biomechanics more similar to healthy controls, implying that immersive VR might assist in diminishing maladaptive movement patterns by redirecting attentional focus from conscious motor control. Moreover, the observed improvements in knee biomechanics suggest that VR training engages neural circuits responsible for motor planning and proprioception, fostering more efficient neuromuscular coordination, as proved by the study of Gokeler et al. [95]. VR can also effectively address the psychological challenges associated with ACLR recovery, particularly the fear of reinjury. By allowing patients to practice challenging movements in a safe and controlled virtual environment, VR can help them overcome their fear and build confidence in their ability to return to sport. This reduction in anxiety can significantly improve overall psychological well-being and facilitate a smoother return to full activity [96]. Another powerful tool that leverages neuroplasticity and motor learning is biofeedback. It represents a modern intervention method that can extensively enhance ACL rehabilitation by providing immediate physiological feedback for the patients [97]. This technique utilizes sensors to monitor muscle activity, joint positioning, and movement patterns, hence aiding athletes in developing a heightened awareness of neuromuscular performance [98]. The integration of biofeedback into rehabilitation programs enables clinicians to help patients regain motor control, correct compensatory movement patterns, and optimize muscle activation, all critical in reducing the risk of reinjury. One of the most significant benefits of biofeedback is the improvement in proprioception and neuromuscular control. For instance, EMG biofeedback might enable the patient to monitor his or her muscle activation level and thereby assist the patient in activating the appropriate muscle groups during rehabilitation exercises [99]. This might be especially beneficial for ACLR patients because quadriceps inhibition and hamstring activation deficit have been commonly demonstrated post-operatively, which may have adverse implications on knee stability and movement efficiency [100,101,102]. Biofeedback EMG offers patients immediate feedback that teaches the correct way to engage those muscles, thereby improving joint stabilization and dynamic control in extreme activities. Moreover, force plate biofeedback systems allow patients to observe weight distribution and balance while performing functional activities like squatting, lunging, or maintaining a single-leg stance [103]. ACLR patients frequently show uneven loading patterns caused by fear of reinjury and muscle imbalances, increasing their risk of secondary injuries. Through the application of biofeedback to address these imbalances, patients can gradually regain normal weight-bearing mechanics, enhancing their confidence and movement efficiency. This new paradigm points out how neuroplasticity exploitation improves outcomes and reduces the risk of reinjury. By integrating cutting-edge rehabilitation techniques, such as robotic-assisted treatments, VR-based therapies, and biofeedback, clinicians can develop a more tailored, data-informed method that enhances neuromuscular rehabilitation.

### 3.2. Long-Term Impact vs. Practical Limitations: Evaluating Advanced Rehabilitation Technologies in ACL Recovery

Importantly, while initial improvements are often observed with these technologies, the long-term efficacy, specifically the sustained and maintained efforts, remains a critical area of investigation. Studies exploring the longitudinal impact of these modalities are increasingly vital to understand if the benefits endure beyond the immediate rehabilitation phase. For instance, a study focusing on biofeedback, using augmented neuromuscular training with real-time visual feedback in young, physically active females, demonstrated significant reductions in peak knee abduction moment after six weeks, a key biomechanical factor in ACL injury risk. This was accompanied by notable increases in functional connectivity between specific brain regions, indicating neural adaptations associated with improved motor control. Furthermore, the degree of biomechanical improvement correlated with increased connectivity between the cerebellum and thalamus, as demonstrated by the study of Diekfuss et al. [104]. It is crucial to acknowledge that although these technologies provide notable benefits, they also have limitations, especially in ACL rehabilitation. For example, robotic systems offer accurate control for activities such as regulated knee extensions or flexion patterns, but they can be costly and necessitate specialized knowledge. Their inflexible design may also struggle to completely mimic the dynamic, multiplanar motions required for sport-specific agility exercises, possibly impeding the athlete’s return to competition [105]. There is also the risk of over-dependence, where an athlete may rely too much on the machine, hindering the growth of independent neuromuscular control crucial for dynamic sports [106]. Likewise, VR can be captivating for activities such as simulated cutting techniques or jump-landing situations, but it may cause motion sickness in certain people, restricting session length and effectiveness. The authenticity of virtual settings may also fail to capture the unpredictable aspects of real-game scenarios, which could influence the application of acquired skills in actual situations [107]. Biofeedback also necessitates patient focus and skilled analysis of data, with its success depending on the clinician’s direction. For instance, although EMG biofeedback can be crucial in retraining quadriceps engagement during single-leg squats, proper sensor placement and interpreting nuanced muscle activation patterns require specialized expertise [108]. Consequently, a measured strategy is essential in incorporating these technologies into ACL rehabilitation, making sure that their drawbacks are thoughtfully considered alongside their advantages to enhance patient care and enable a secure and efficient comeback to sports. Table 1 provides an overview of advanced rehabilitation techniques in ACL recovery, highlighting the integration of innovative technologies such as robotics, VR, and biofeedback to enhance outcomes through neuroplasticity mechanisms.

## 4. Cerebral Compensation: How Neuroplasticity Shapes ACLR Recovery Beyond the Knee

Neuroplasticity-driven recovery in ACLR reaches well beyond localized rehabilitation at the level of the knee, with diffuse adaptations in numerous brain regions involved in motor control, proprioception, and cognitive–motor integration, as we discussed earlier. Following a tear in the ACL, afferent perturbation of sensation generates maladaptive reorganizations in the sensorimotor network, most prominently in the primary motor cortex (M1), with its changed excitability and impaired corticospinal drive to the quadriceps [109]. Reorganization in the cortex generates compensatory activation profiles, defaulting to ipsilateral hemisphere motor control or secondary motor regions, including the supplementary motor area (SMA) and premotor cortex, in an attempt at compensating for loss in automatized performance of motor function [110,111,112]. The cerebellum, a crucial structure for movement precision and error correction, also undergoes significant alterations. ACL injury compromises cerebellar contributions to motor adaptation, leading to delayed or inefficient adjustments in joint stabilization strategies. This may explain the persistent difficulties in dynamic balance and reactive postural control observed in ACLR patients, even after regaining strength [113]. The integration of cerebellar-driven motor learning is therefore essential to recalibrate movement patterns and prevent compensatory strategies that could predispose individuals to further injury. The study of Schnittjer et al. found that individuals with ACLR exhibit distinct neuroplastic adaptations in the cerebellum and sensorimotor cortex during knee motor control. Specifically, brain activity between ACLR patients and healthy controls significantly overlapped in both the sensorimotor cortex and cerebellum. However, compared to controls, the ACLR group demonstrated unique cortical activations, particularly in the superior parietal regions (precuneus, lateral occipital cortex) and a shift in peak activation in the frontal and parietal regions. These findings suggest that ACLR leads to a compensatory, cortically driven sensory integration strategy to maintain motor control of the affected limb, independent of strength, self-reported knee function, or time since surgery [114]. Another critical area affected by ACL injury is the somatosensory cortex, where deficits in proprioceptive processing contribute to diminished knee joint awareness. Functional magnetic resonance imaging (fMRI) studies reveal that ACLR patients exhibit reduced activation in this region during joint position tasks, underscoring the necessity of sensory reweighting strategies in rehabilitation [115]. For instance, Criss and his team found through fMRI that people who report better knee function show heightened activity in the ipsilateral secondary somatosensory cortex and the SMA when performing knee movement tasks [116]. This suggests that enhanced engagement of these brain regions may serve as a compensatory mechanism to preserve motor control and proprioceptive integration following ACL injury and reconstruction. By targeting proprioceptive training through unstable surfaces, external perturbations, and augmented feedback systems, clinicians can facilitate the reintegration of accurate sensory information into movement planning (specifically motor planning) [117].

### Brain Activity and Kinesiophobia: Understanding the Neurological Impact of ACLR

Beyond motor and sensory domains, the prefrontal cortex plays a pivotal role in ACLR recovery by influencing movement anticipation, risk assessment, and fear modulation [118]. Heightened activity in this region is often observed in patients exhibiting fear of reinjury, suggesting a cognitive overload that interferes with automatic motor execution [119,120]. Individuals with ACLR may exhibit diminished brain activity in the right ventrolateral prefrontal cortex during the action-observation drop vertical jump task [121,122]. In addition, the brain activity of ACLR patients in regions such as the left cerebellum Crus I and II, right cerebellum lobule IX, amygdala, middle temporal gyrus, and temporal pole demonstrated a positive relationship with higher kinesiophobia scores, assessed by the Tampa Scale of Kinesiophobia (TSK-11) as demonstrated by the study of Kim and colleagues [121]. These findings suggest that alterations in brain activity within regions associated with fear and cognitive processes, such as the prefrontal cortex and areas related to emotional regulation, are linked to the fear of movement in patients who have had ACLR. This maladaptive hypervigilance can lead to suboptimal movement strategies, such as over-reliance on visual feedback or excessive muscle co-contraction, which may ultimately increase the risk of secondary injury [123]. Addressing these cognitive–motor interactions through dual-task training and immersive rehabilitation environments, such as VR, can help normalize prefrontal engagement and restore fluid, automatic movement control. This method is backed by research showing the effectiveness of immersive rehabilitation in reducing kinesiophobia. For instance, a study assessing a cognitive–behavioral multimedia program in patients undergoing ACL surgery showed that those using the program expressed increased preoperative coping confidence and notably less postoperative kinesiophobia compared to individuals receiving standard treatment [124]. This implies that engaging, immersive programs can successfully tackle the mental elements of recovery, especially the anxiety of reinjury. Further supporting this idea, a randomized controlled trial examining VR in conjunction with exercise in female patients post-total knee arthroplasty also revealed a significant decrease in kinesiophobia among the VR group [125]. This research emphasized that immersive VR interventions can effectively reduce movement-related fear, leading to decreased pain levels and better functional results, as shown by improved knee flexion and performance in functional assessments. Although the use of immersive rehabilitation to alleviate kinesiophobia is becoming more popular, these studies highlight its possible role as an important element in complete rehabilitation strategies, indicating that it is not completely new but rather an evolving field with encouraging clinical uses.

## 5. Overcoming Persistent Deficits: Addressing Sensory, Neuromuscular, and Muscular Challenges in ACL Rehabilitation

**Figure 3:**
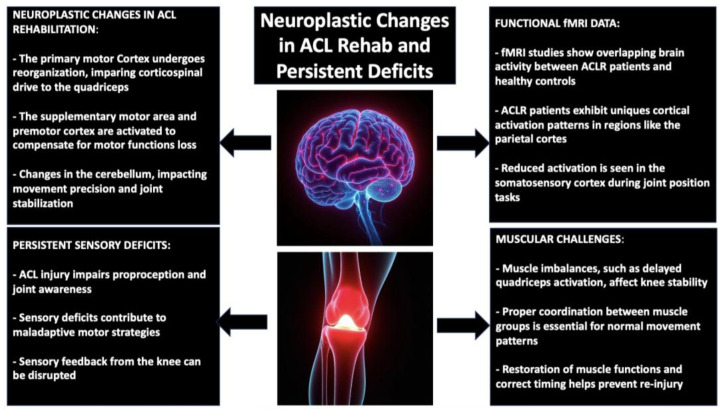
Neuroplastic changes in ACL rehab and sensory and neuromuscular deficits.

A deeper exploration of the persistent challenges in ACL rehabilitation requires an understanding of how sensory deficits, muscle imbalances, and neurological adaptations collectively contribute to the prolonged recovery faced by ACLR patients. While traditional rehabilitation often prioritizes muscle strength recovery, deficits in neuromuscular control, proprioception, and sensory integration may linger for months or even years, hindering functional restoration and elevating the risk of reinjury. The sensory function of the ACL, specifically its role in proprioception, plays a crucial part in the rehabilitation process. Following ACL injury, the ligament’s ability to detect joint position and movement is compromised, leading to deficits in joint awareness and motor coordination [126]. These proprioceptive impairments are linked to maladaptive motor strategies that hinder the development of normal movement patterns, leaving the knee vulnerable to injury [127]. As mentioned, rehabilitation strategies must go beyond addressing muscle strength alone; they should also focus on restoring the sensory feedback loop from the knee to the brain. Incorporating exercises that challenge proprioception, such as standing on one leg with eyes closed, perturbation training, and dynamic balance exercises, can gradually restore the sensory function of the knee and reduce the risk of reinjury. Additionally, research underscores that the deficits associated with ACLR can persist long after surgical intervention. Studies by Tayfur et al. emphasize the interaction of muscular and neural factors in post-ACL repair rehabilitation. The loss of muscle size and function is compounded by a diminished sensory role of the ACL, which plays a critical role in proprioception [128]. While structural stability is a major focus of ACLR, restoring the sensory function of the knee is equally important for long-term success and preventing reinjury. It is important to challenge balance by standing on one leg and hopping, improve proprioception with closed-eye exercises and perturbation training, and enhance neuromuscular control with plyometrics and sport-specific drills to restore sensory functions. These results highlight the need to focus on rehabilitation protocols not only for muscle and neural recovery [129]. Besides focusing on sensory deficits and neuromuscular control, another essential aspect that needs attention in ACL rehabilitation is the correction of muscle imbalances. Following an ACL injury and the resulting reconstruction, the quadriceps, hamstrings, and calf muscles frequently demonstrate changed recruitment patterns, with the quadriceps usually exhibiting delayed activation. This muscle imbalance impacts not just the leg’s total strength but also affects the stability and functional ability of the knee joint [130]. Studies show that enhancing the hamstrings alongside the quadriceps can aid in re-establishing balance, lowering the chances of reinjury, and boosting knee joint stability [131,132]. Nonetheless, this involves not only enhancing muscle strength but also reinstating correct timing and coordination among the muscle groups to bring back normal movement patterns. Additionally, focus should be placed on the overall biomechanical alignment of the joint. Irregular joint mechanics, frequently arising from postural adjustments or insufficient neuromuscular coordination, can heighten the risk of reinjury and extended functional impairments. Gait retraining, combined with exercises that focus on correct knee alignment when weight-bearing, can help in recovering proper mechanics and minimizing abnormal stress on the joint. Methods like step-up exercises, squats, and lunges with accurate knee tracking are crucial for enhancing joint alignment and advancing movement patterns that aid in long-term recovery [133]. In the future, leveraging innovative technologies and interdisciplinary collaboration will be crucial for creating highly personalized and powerful rehabilitation programs. This approach is essential for maximizing long-term success rates and minimizing reinjury risk. Figure 3 illustrates the neuroplastic changes during ACL rehabilitation and highlights the persistent sensory and neuromuscular deficits that continue to impact recovery long after surgery.

### The Next Frontier in ACL Rehabilitation: Integrating Innovation and Expertise

**Figure 4:**
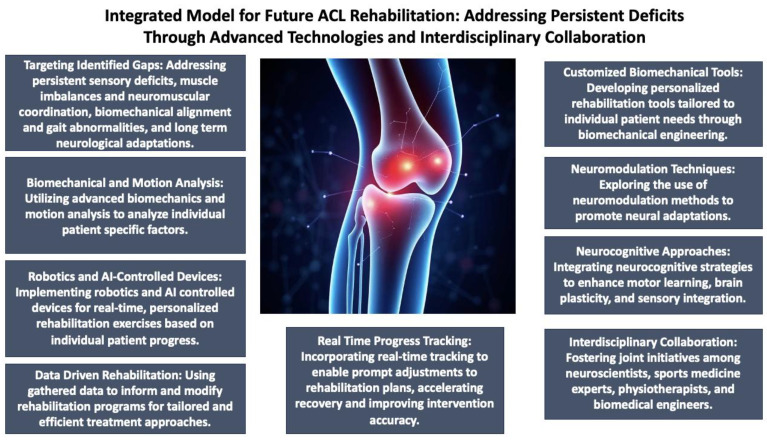
Integrated model for future ACL rehabilitation.

The future of ACL rehabilitation, however, must be targeted towards filling these gaps identified (persistent sensory deficits, muscle imbalances and neuromuscular coordination, biomechanical alignment and gait abnormalities, and long-term neurological adaptations). Developments in biomechanics and motion analysis offer effective resources for the analysis of the individual factors specific to a patient. When specific neural impairments are identified, rehabilitation practitioners can adapt interventions to target these impairments more directly [134]. Also, by means of robotics and AI-controlled devices, one can envision increasingly tailor-made rehabilitation courses of exercises in real-time, according to the patients’ individual improvement [135,136]. Furthermore, longitudinal monitoring presents a valuable chance to improve results in ACL rehabilitation through ongoing evaluation of the recovery journey. Establishing systems that monitor functional performance, including joint mobility, strength, and movement patterns, along with reinjury rates across prolonged durations, can result in a deeper comprehension of the complexities inherent in patient recovery [137]. Using this information, healthcare practitioners can make well-informed choices regarding modifications to rehabilitation programs, thereby guaranteeing more tailored and efficient treatment approaches. Gathering these extensive data would facilitate the improvement of current best practices and aid in creating evidence-based strategies customized to specific requirements [138]. As this knowledge base develops over time, it can diminish the frequency of reinjuries and enhance functional outcomes, which is crucial for successful long-term rehabilitation. Moreover, incorporating real-time tracking of progress might allow for prompt adjustments to the rehabilitation plan, thereby speeding up recovery and improving the accuracy of interventions [139]. Additionally, an authentic interdisciplinary approach to ACL rehabilitation is essential and can significantly change the future of care. Joint initiatives involving neuroscientists, sports medicine experts, physiotherapists, and biomedical engineers are essential for tackling the intricate and diverse aspects of ACL rehabilitation [140]. By utilizing the knowledge from these various domains, creative solutions can be created that not only tackle the physical components of recovery but also integrate neurocognitive approaches to improve motor learning, brain adaptability, and sensory unification [141]. For example, neuromodulation methods might be incorporated into rehabilitation programs to enhance neural changes, while biomechanical engineers could create more customized rehabilitation tools suited to specific patients’ requirements [139]. This comprehensive model could transform ACL rehabilitation, advancing past conventional methods to connect innovative scientific research with clinical practice. By developing more personalized, multidimensional interventions, it is possible to offer a more holistic recovery process that prioritizes both the body and mind, ensuring improved long-term outcomes for patients. Figure 4 visually represents an integrated model for future ACL rehabilitation.

## 6. Discussion: Expanding Neurorehabilitation Insights into Sports Medicine

This investigation on ACL rehabilitation uncovers a fascinating intersection of sports medicine, neuroscience, and cutting-edge technology. It is clear that repairing an isolated ligament alone is inadequate; the injury triggers a complicated sequence of reactions impacting the whole neuromuscular system, reaching cortical levels. Traditional methods, mainly focusing on strength and flexibility, while useful, frequently do not sufficiently tackle ongoing deficiencies in proprioception, neuromuscular control, and the psychological effects of injury. These persistent difficulties greatly hinder an athlete’s comeback to their sport and increase the likelihood of reinjury. A shift in paradigm towards a rehabilitation strategy centered on neuroplasticity presents significant transformative potential. The idea of reprogramming the brain, restructuring neural connections, and enhancing brain–knee interaction offers new treatment possibilities. Neuromodulation methods, including transcranial magnetic stimulation (TMS) and transcutaneous electrical nerve stimulation (TENS), show potential in rehabilitation environments [142,143,144,145,146,147]. In particular, TMS has the ability to stimulate specific cortical areas that are associated with motor control and proprioception, which may improve neuroplasticity and speed up motor learning [148]. TENS can alleviate pain and lessen muscle spasms, enabling better engagement in rehabilitation exercises [149]. These non-invasive methods provide a way to directly influence neural activity and enhance the brain’s receptiveness to rehabilitative treatments. The advancement of advanced wearable sensors and implantable devices offers clinicians remarkable insight into the recovery path of an athlete. These technologies facilitate ongoing observation of joint movement, muscle activation, heart rate variability, and sleep quality, providing a thorough evaluation of physiological and neurological conditions [150]. This data-centric method enables customized rehab plans, objective monitoring of progress, and early identification of fatigue or overtraining, allowing for prompt modifications to reduce injury risk [151,152]. In contrast to traditional methods, robotics can aid in providing precise, regulated movements, as previously discussed [153]. This can assist in muscle recovery and the re-acquisition of motor skills. Additionally, the incorporation of artificial intelligence (AI) and machine learning algorithms could transform rehabilitation assessment, tracking, and personalization [154]. AI algorithms are capable of examining large datasets obtained from wearable sensors, motion capture technologies, and self-reported patient outcomes to detect subtle trends and forecast the risk of reinjury [155,156]. This data-focused method enables clinicians to make knowledgeable choices about the timing and intensity of activities, enhance rehabilitation plans in real-time, and forecast the likelihood of returning to sport successfully. The incorporation of technology should be combined with a greater comprehension of the neurocognitive aspects of recovery. It is essential to tackle the fears of reinjury, anxiety, and psychological obstacles that hinder progress [157]. This requires integrating psychological assistance, mindfulness practices, and strategies for building resilience into the rehabilitation process [158]. In addition, promoting interdisciplinary collaboration among sports medicine experts, neuroscientists, biomechanical engineers, and psychologists is crucial for creating thorough and personalized rehabilitation programs [159]. The progression of ACL rehabilitation relies on utilizing neuroplasticity, adopting technological advancements, and fostering interdisciplinary teamwork [160]. By cohesively combining these components, we can elevate individual knee therapies and enhance the whole sensorimotor system, allowing athletes to not just recover but also excel. This change in approach has the potential to revolutionize ACL rehabilitation and improve the treatment of various musculoskeletal injuries, ultimately resulting in better outcomes and greater functional recovery for athletes at all performance levels.

## 7. Conclusions

Neuroscientific integration in sports rehabilitation represents a watershed moment in the process of recovery. We are advancing past a solely structural perspective of the body, acknowledging the essential role of the nervous system, mind, and brain in the healing process. By utilizing the concepts of neuroplasticity, rehabilitation experts can tackle the intricate interactions of physical and neurocognitive elements that affect results following ACL injuries. Technological innovations and interdisciplinary collaboration will continue advancing and optimizing rehabilitation protocols. The integration of real-time data analytics, immersive VR, and robotics will further the process of innovation, which will facilitate more accurate and patient-specific care. In addition, adherence to longitudinal follow-up and evidence-based interventions will guarantee that the discipline keeps up with new challenges and opportunities. Finally, linking the dots between neuroscience and sports medicine opens the road toward a common platform that emphasizes the well-being of the athlete. Through the crossing of these fields, the discipline can map a course for more efficient, integrative, and lasting recovery models that not only reduce reinjury risk but also maximize long-term recovery.

## Figures and Tables

**Table 1:** Overview of advanced rehabilitation techniques in ACL recovery.

Rehabilitation Focus	Definition	Key Mechanism	Rehabilitation Approach	Impact on ACL Recovery	Practical Application	Limitations of the Advanced Techniques.
Neuroplasticity in ACL Rehab [15,17,18]	The brain’s ability to reorganize and form new neural connections after injury.	Reorganization of neural pathways and motor skills.	Incorporating proprioceptive and cognitive training alongside physical exercises.	Enhances recovery by restoring communication between knee and brain, improving movement patterns.	Using complex exercises combining balance and strength to enhance brain–knee communication.	Requires high patient engagement and consistency; neural adaptation varies between individuals.
Proprioception and Sensory Feedback [80,81]	The ability to detect joint position and movement, essential for coordination and injury prevention.	Activation of sensory receptors near the knee, promoting brain-knee communication.	Exercises on unstable surfaces or tasks requiring precise movements.	Restores knee stability and reduces risk of future injuries.	Balance exercises on a Bosu ball or wobble board to improve joint awareness.	May not be suitable for all rehabilitation stages; risk of instability-related setbacks.
Cognitive Function and Decision-Making [70]	Cognitive function involves mental processes like attention, memory, and problem-solving, essential for making quick, informed decisions, especially under pressure. Effective decision-making in athletes relies on these cognitive abilities, influencing performance and injury prevention.	Cognitive load (e.g., multi-tasking or unexpected stimuli) improves decision-making and reaction time.	Incorporating tasks like counting during drills or responding to unpredictable stimuli.	Improves reaction speed and confidence, reducing reinjury risk during dynamic sports situations.	Drills that require athletes to respond to unexpected movements or make quick decisions during rehabilitation.	High cognitive demands may increase mental fatigue; effectiveness depends on individual cognitive ability.
Robotic-Assisted Rehabilitation [89,90]	Robotic devices help in executing precise, regulated movements to aid muscle recovery.	Exact biomechanical movements to improve motor function and neuromuscular control.	Use of exoskeletons or robotic trainers to guide exercises and monitor progress.	Enhances recovery through muscle strengthening, joint stability, and motor skill re-learning.	Robotic-assisted walking devices to improve gait and functional movements after ACL reconstruction.	High cost and limited accessibility; may not replicate sport-specific dynamic movements.
VR Training [93,94]	Immersive virtual environments provide scenario-based training to improve movement control.	Induces neural changes for better coordination, proprioception, and knee biomechanics.	VR-based drills simulating unpredictable sports movements.	Improves knee biomechanics and psychological readiness for return to sport, reducing fear of reinjury.	VR simulations of game-like scenarios (e.g., basketball pivoting) to prepare athletes for return to play.	Requires specialized equipment; limited real-world transferability for some movement patterns.
Biofeedback Systems [96,97,98,99]	Provides real-time feedback on muscle activity and movement patterns to aid recovery.	Monitoring of muscle activity, joint position, and weight distribution.	Use of EMG and force plate biofeedback to guide muscle activation and balance.	Improves proprioception, neuromuscular control, and corrects compensatory movements, reducing reinjury risk.	EMG biofeedback to monitor and enhance quadriceps activation post-surgery to promote better knee stability.	Requires specialized training and equipment; effectiveness depends on patient compliance and proper use.

Legend: anterior cruciate ligament (ACL), virtual reality (VR), electromyography (EMG).
